# Transcriptome analysis reveals that jasmonic acid biosynthesis and signaling is associated with the biosynthesis of asperosaponin VI in *Dipsacus asperoides*

**DOI:** 10.3389/fpls.2022.1022075

**Published:** 2022-12-22

**Authors:** Jiao Xu, Zhengping Hu, Hua He, Xiaohong Ou, Yang Yang, Chenghong Xiao, Changgui Yang, Liangyuan Li, Weike Jiang, Tao Zhou

**Affiliations:** Resource Institute for Chinese Medicine and Ethnic Materia Medica, Guizhou University of Traditional Chinese Medicine, Guiyang, China

**Keywords:** *Dipsacus asperoides*, asperosaponin VI, transcriptome, jasmonic acid, triterpenoid biosynthesis

## Abstract

*Dipsacus asperoides* is a perennial herb, the roots of which are abundant in asperosaponin VI, which has important medicinal value. However, the molecular mechanism underlying the biosynthesis of asperosaponin VI in *D. asperoides* remains unclear. In present study, a comprehensive investigation of asperosaponin VI biosynthesis was conducted at the levels of metabolite and transcript during root development. The content of asperosaponin VI was significantly accumulated in two-leaf stage roots, and the spatial distribution of asperosaponin VI was localized in the xylem. The concentration of asperosaponin VI gradually increased in the root with the development process. Transcriptome analysis revealed 3916 unique differentially expressed genes (DEGs) including 146 transcription factors (TFs) during root development in *D. asperoides*. In addition, α-linolenic acid metabolism, jasmonic acid (JA) biosynthesis, JA signal transduction, sesquiterpenoid and triterpenoid biosynthesis, and terpenoid backbone biosynthesis were prominently enriched. Furthermore, the concentration of JA gradually increased, and genes involved in α-linolenic acid metabolism, JA biosynthesis, and triterpenoid biosynthesis were up-regulated during root development. Moreover, the concentration of asperosaponin VI was increased following methyl jasmonate (MeJA) treatment by activating the expression of genes in the triterpenoid biosynthesis pathway, including acetyl-CoA acetyltransferase (*DaAACT*), 3-hydroxy-3-methylglutaryl coenzyme A synthase (*DaHMGCS*), 3-hydroxy-3-methylglutaryl coenzyme-A reductase (*DaHMGCR*). We speculate that JA biosynthesis and signaling regulates the expression of triterpenoid biosynthetic genes and facilitate the biosynthesis of asperosaponin VI. The results suggest a regulatory network wherein triterpenoids, JA, and TFs co-modulate the biosynthesis of asperosaponin VI in *D. asperoides*.

## Introduction

**Figure 1:**
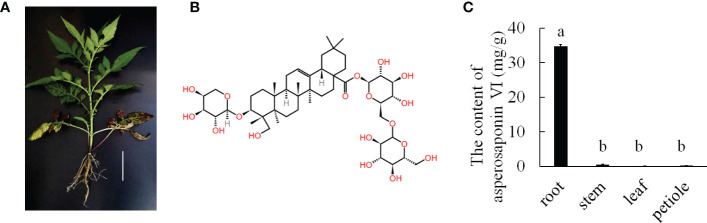
The major medicinally active triterpenoid in *D. asperoides*. **(A)**, the plant of *D. asperoides*. Scale bar = 10 cm. **(B)**, the typical triterpenoid of asperosaponin VI. **(C)**, the content of asperosaponin VI in different tissues of *D. asperoides*. The error bars represent the standard deviation with at least three biological replicates. Different lowercase letters denote significant differences by multiple comparisons using Statistix 8.0 software.

*Dipsacus asperoides* is a perennial herb belonging to the Dipsacaceae family (**Figure 1A**). It is a Chinese herbal medicinal that is used for the treatment of bone fractures and joint diseases such as osteoporosis, lassitude in the loin and legs, and fractures by preventing bone loss, increasing osteoblastic activity, and decreasing osteoclastic activity (Niu et al., 2012; Niu et al., 2015). Notably, asperosaponin VI is the main active component of *D. asperoides*, which is an oleanane triterpenoid saponin. In addition, asperosaponin VI may increase bone formation by increasing BMP-2 synthesis and activating both p38 and ERK1/2 (Niu et al., 2011). Asperosaponin VI may be a potential agent for suppressing neuroinflammation related to Alzheimer’s disease (Yu et al., 2012). Moreover, because it activates the PI3K/Akt and CREB pathways, asperosaponin VI has protective effects against hypoxia-induced cardiomyocytes apoptosis (Li et al., 2010). Therefore, *D. asperoides* has very important medicinal value because of its content of asperosaponin VI.

Asperosaponin VI is a triterpenoid saponin, and thus it is biosynthesized *via* the mevalonic acid (MVA) pathway which has been found in previous studies (Yang et al., 2017; Chen et al., 2018; Yang et al., 2018). Typically, the pathway involves the conversion of isoprenyl diphosphate (IPP) and dimethylallyl diphosphate (DMAPP) into farnesyl pyrophosphate (FPP), which then generates squalene and 2,3-oxidosqualene, and is followed by a series of reactions involving cyclization, oxidation, hydroxylation and glycosylation (Kuzuyama, 2002; Augustin et al., 2011; Niu et al., 2014), which is accompanied by a variety of enzymatic reactions involving various enzymes such as farnesyl pyrophosphate synthase (FPPS), β-amyrin synthase (β-AS), and dammarenediol synthase (DS). There are great differences in the quality of *D. asperoides* from different production areas, and the content of asperosaponin VI also varies greatly due to great differences in the environment, such as altitude, temperature, and light conditions (Jiang et al., 2013). Moreover, germplasm resources differ greatly in different producing areas, resulting in different qualities of *D. asperoides* (Xiao et al., 2018). Previous studies have illustrated that the genes encoding asperosaponin VI synthetase are involved in the triterpenoid biosynthesis pathway (Wang et al., 2016). However, the genes belong to multigene families that are complex and diverse. To date, little is known about the genes functioning in triterpenoid biosynthesis in *D. asperoides*. Additionally, the mechanisms of its biosynthesis and regulation are unclear. Hence, analyzing the biosynthesis pathway of triterpenoid saponins and verifying the gene function are important.

To explore the biosynthesis pathway of triterpenoid saponins, omics methods have been conducted in recent years. The genomes of *P. notoginseng* and *P. ginseng* have been sequenced. UDP-glycosyltransferases, which belong to one of the largest gene families, catalyze the formation of different ginsenosides. Five glycosyltransferase genes were identified to catalyze the formation of different ginsenosides in *P. notoginseng* (Xu et al., 2017; Jiang et al., 2020). Other than glycosyltransferase genes, oxidosqualene cyclases, and cytochrome P450 are also involved in the biosynthesis of triterpenoid saponins (Yang et al., 2021). Moreover, transcriptome analysis has already been applied to investigations on terpenoid metabolism (Garg et al., 2015; Fan et al., 2019). Plant growth conditions play important roles in the biosynthesis of triterpenoid saponins. Research shows that more gibberellins and steroids accumulate in the root with quick vegetative growth under favorable conditions, and abscisic acid (ABA) and ginsenosides also accumulate in the root to enhance resistance under abiotic stress. In a functional study using transgenic *Arabidopsis*, terpene synthase genes were expressed and terpenoid contents increased compared with the wild-type control. Transcriptome and metabolome data showed the complexity of metabolic genes in terpenoid-rich *Salvia guaranitica* (Ali et al., 2018). With respect to asperosaponin VI, transcriptome analysis is urgently required to reveal the mechanism of regulation and biosynthesis of asperosaponin VI in *D. asperoides*. In addition, it is urgent to focus on molecular breeding to address the quality issue in *D. asperoides*.

In this study, matrix-assisted laser desorption ionization-mass spectrometry imaging (MALDI-MSI) was used to observe the spatial distribution pattern of asperosaponin VI. The roots at different stages of development were selected for detecting the content of asperosaponin VI. Three periods L1, L2 and M7 with large differences in asperosaponin VI content were selected for transcriptome sequencing by Illumina sequencing technology. Gene Ontology (GO) analysis, Kyoto Encyclopedia of Genes and Genomes (KEGG) pathway analysis, and methyl jasmonate (MeJA) treatment were used to identify the gene expression and regulation signaling, thereby revealing new mechanistic insights into asperosaponin VI biosynthesis in *D. asperoides*.

## Materials and methods

### Plant materials and treatments

The *D. asperoides* plants were cultivated in March at Guizhou University of Traditional Chinese Medicine, Guizhou Province, China. The roots of one-leaf stage (L1), two-leaf stage (L2), and four-leaf stage (L4) plants, 7 months (M7) and 12 months (M12), as well as roots, leaves, stems, and petioles from plants that had been growing for approximate 2 years were frozen immediately in liquid nitrogen and stored in a −80 °C refrigerator for RNA isolation, JA and asperosaponin VI detection. The seedlings were cultured in ½ Murashige & Skoog (MS) solution under 16 h light/8 h dark conditions at 25 °C for MeJA treatment.

### MALDI MSI and data analysis

Whole plants of *D. asperoides* were dug out from the field. The experiment was performed as previously described (Sun et al., 2021). The roots of *D. asperoides* were flash-frozen in liquid nitrogen for 15 s and then was transferred to a −80°C refrigerator. The root tissues were cryo-sectioned into 20-μm cross sections at −20°C on a cryostat microtome (Thermo CryoStar NX50 NOVPD) and mounted onto a conductive side of indium tin oxide (ITO)-coated glass slide. Root sections from different periods were prepared for MALDI matrix optimization. All these root sections were then transferred to a closed container and vacuum dried for 10 min. Then 10 mg/mL of DHB (2,5-Dihydroxybenzoic acid) in ACN/H_2_O/TFA (Acrylonitrile/H_2_O/trifluoroacetic acid, 70:30:0.1, v/v/v) was selected as the matrix. Matrix coating was performed on a HTX TM-Sprayer™ (HTX Technologies). The flow rate of the sprayer was set to 0.075 mL/min at 55°C. The track speed was set to 800 mm/min and the track spacing was 3 mm. MALDI-MSI imaging was conducted on a Rapiflex MALDI Tissue Typer TM TOF/TOF MS (Bruker Daltonics). The laser was fired at a repetition rate of 5000 Hz, and the spatial resolution was set to 100 μm. Raw mass spectra data were acquired over the *m/z* range of 80–1200. Raw mass spectra were imported into Data Analysis 4.0 software (Bruker Daltonics) to perform internal mass calibration. The MS images were viewed and processed using SCiLS Lab 2018b (GmbH) software.

### Paraffin section analysis

The roots of seedlings at the L2 stages were fixed in 50% FAA (Formalin-Aceto-Alcohol) as described previously (Hou and Huang, 2005). And the sections stained with hematoxylin solution and eosin dye.

### Extraction and detection of asperosaponin VI

Asperosaponin VI was extracted from the roots of the *D. asperoides* samples with methanol according to our previous method with at least three independent biological replicates (Jiang et al., 2013). Approximately 0.1 g dried root powder was mixed with 5 mL methanol and extracted with ultrasonic treatment at 59 kHz for 30 min. Then the supernatant was collected after centrifugation at 4000 rpm for 10 min and filtered through a 0.45 µm filter. The standard of asperosaponin VI was dissolved in methanol and then mixed and diluted with methanol to obtain a series of mixture standard solutions of different concentrations. The solutions were filtered through a 0.45 µm syringe filter before high-performance liquid chromatography (HPLC) analysis.

Samples were separated on a Wondasil C18 column (4.6 mm × 250 mm, 5 μm) and detected at 212 nm. The column temperature was maintained at 30°C, and the flow rate was 1 mL/min. The mobile phase consisted of 30% acetonitrile. Asperosaponin VI was used as a standard to determine the asperosaponin VI content of the *D. asperoides* tissue.

### RNA isolation and RNA sequencing

Nine samples at different stages of development were used for RNA sequencing, including L1, L2, M7 with three biological replicates. Total RNA was extracted following the instructions of Eastep^®^ Super Total RNA Extraction Kit (Promega). The integrity and quality of the RNA were assessed using RNA agarose gel electrophoresis and NanoDrop2000 (Thermo Fisher Scientific). The RIN value was determined by the RNA 6000 Nano Kit of an Agilent Bioanalyzer 2100 (Agilent Technologies).

The cDNA libraries and the RNA-Seq were performed at Shanghai Majorbio Bio-pharm Technology Co., Ltd. (Shanghai, China). The mRNA was purified using oligo (dT) magnetic beads. Taking these short fragments as templates, double-stranded cDNA was synthesized by a SuperScript double-stranded cDNA synthesis kit (Invitrogen). Libraries were size selected for cDNA target fragments of 200~300 bp. After quantification by TBS380, nine RNA-Seq libraries were sequenced by an Illumina NovaSeq 6000 sequencer (Illumina) for 2×150 bp paired-end reads. About 6 Gb reads were obtained from each sample for *de novo* assembly.

### *De novo* assembly and annotation

To obtain high-quality clean data to ensure the success of subsequent analyses, the data quality control was used by FastQC, the raw reads with N ratios exceeding 10%, low-end sequence bases (mass values less than 20), or sequences with adaptors were all filtered using fastx_toolkit Version 0.0.14 (http://hannonlab.cshl.edu/fastx_toolkit/). Then all clean reads were used for *de novo* assembly with Trinity (http://trinityrnaseq.sourceforge.net/). Afterward, the TPM was calculated by RSEM (http://deweylab.github.io/RSEM/software). Furthermore, all transcripts were compared with the Nr (NCBI Non-redundant Protein Library), String (Public Member Function), Swiss-Prot (Swiss-Prot protein database), Pfam (Protein Family Database), and KEGG databases using Blastx to obtain corresponding annotation information. To identify putative transcription factors (TFs), the Blastx was performed against PlantTFDB (http://planttfdb.cbi.pku.edu.cn/) (Jin et al., 2017).

### Analysis of differentially expressed genes

The differentially expressed genes (DEGs) were analyzed by comparing the Fragments Per Kilobase of transcript per Million mapped reads (FPKM) of the unigenes in the L1, L2, and M7 samples. Based on padj <0.05, absolute value |log_2_ fold change| > 2, and filtering according to (FPKM) max > 30, the unigenes were considered to be significantly differentially expressed. All DEGs were subjected to GO enrichment using Blast2GO with a corrected *P-value* < 0.05 (http://www.blast2go.com/b2ghome). GO functional analysis was conducted according previously study (Mishra et al., 2009). The KEGG pathway was used to obtain metabolic pathway analysis with a *P-value* < 0.05.

### Expression correlation analysis

After the genes of interest are obtained, the correlation coefficients between genes are obtained by Pearson correlation algorithm based on the correlation of gene expression, and the visual network diagram was drawn. The data were analyzed on the online tool of Majorbio Cloud Platform (Ren et al., 2022).

### Quantitative real-time PCR analysis

Candidate unigenes from the RNA-Seq were selected and validated by qRT-PCR. The first-strand cDNA was synthesized from 1 μg of total RNA using a cDNA synthesis kit (Promega). qRT-PCR was performed in 20 μL reactions using an Applied Biosystem 7500 real-time PCR system (Applied Biosystems) with at least three independent biological replicates. The gene *DaActin103* was used as the internal control to normalize and calculate the gene expression (Liang et al., 2020). The relative gene expression was calculated using the 2^−ΔCt^ method. The primers used for qRT-PCR are listed in **Supplementary Table S1**.

### Endogenous JA extraction and quantification

To measure the concentration of JA, approximately 0.1g root samples were extracted in 80% cold methanol overnight. Further extraction and quantification analyses of JA were performed as described previously (Liu et al., 2012). The standard JA was purchased from Sigma-Aldrich.

### MeJA treatment

In order to detect the regulation of JA on the biosynthesis of asperosaponin VI in *D. asperoides*, seedlings at the L2 stages were treated with 150 μM MeJA (Hu et al., 2021). The roots were collected following MeJA treatment for 6 h and 5 d, rapidly placed in liquid nitrogen, and frozen at –80 °C for RNA isolation and asperosaponin VI detection.

## Results

### Plant phenotype and asperosaponin VI accumulation in *D. asperoides*

**Figure 2:**
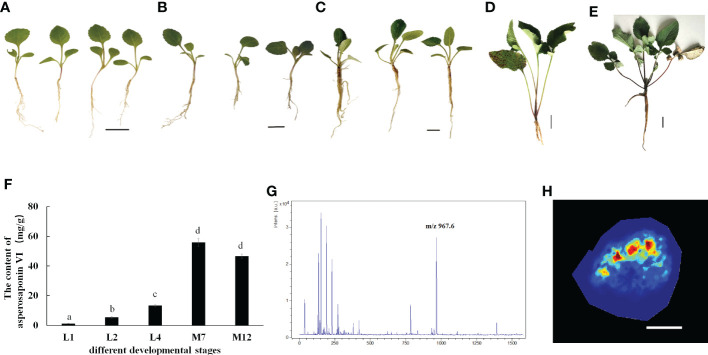
Accumulation Pattern of asperosaponin VI in root. **(A–E)**, the plants of D. asperoides in one leaf stage (L1), two leaf stage (L2), four leaf stage (L4), seven months (M7), one year (12M). Scale bars = 2 cm in **(A–C)**, Scale bars = 5 cm in **(D, E)**. **(F)**, the content of asperosaponin VI in **(A–E)**. The error bars represent the standard deviation with at least three biological replicates. Different lowercase letters denote significant differences by multiple comparisons using Statistix 8.0 software. **(G)**, the asperosaponin VI peak is most abundant (m/z 967). **(H)** Accumulation Pattern of asperosaponin VI in L2 root as observed by MALDI MSI. Scale bars = 1 mm.

*D. asperoides* is a medicinal herb. Our results showed that the roots of *D. asperoides* were rich in asperosaponin VI (**Figure 1B**). The other tissues in the aboveground part of the plant such as the petiole, stem and leaf had very low levels of asperosaponin VI (**Figure 1C**). To monitor the dynamics of asperosaponin VI metabolism, the roots were sampled at different developmental stages (L1, L2, L4, M7, and M12) (**Figures 2A–E**). The results showed that the accumulation of asperosaponin VI gradually increased during development. Only a small amount of asperosaponin VI was detected at the L1 stage, while it was significantly accumulated at the L2 stage. Additionally, it reached a high level at the M7 stage (**Figure 2F**, **Figure S1**). We suspect that asperosaponin VI gradually accumulated along with root development in the early growth stage in *D. asperoides*.

### Spatial distribution pattern of asperosaponin VI

Given the accumulation of asperosaponin VI at initial period in the roots of L2, the roots were sampled to visualize the spatial distribution of asperosaponin VI by MALDI-MSI with a lateral resolution of 100 μm. The molecular ions detected in our study were identified according to the fragmentation of standard substances. The maximum peak area (**Figure 2G**) was identified as asperosaponin VI, which accumulated mainly in the xylem (**Figure 2H**, **Figure S2**). However, less was detected in the other parts. This result further illustrated that asperosaponin VI accumulated in the roots at stage L2, and furthermore, the accumulation of asperosaponin VI was associated with the development of the xylem in the roots of *D. asperoides*.

### RNA sequencing and *De novo* assembly

To explore the mechanism of asperosaponin VI biosynthesis during the developmental stages and study the transcriptional regulatory networks for the biosynthesis of asperosaponin VI in *D. asperoides*, RNA-Seq was performed on *D. asperoides* roots during developmental stages of great difference (L1, L2, M7): the accumulation of asperosaponin VI at the beginning in the roots from L1 to L2, and the content of asperosaponin VI was the highest in the root of M7. Each sample had three biological replicates. Based on Illumina NovaSeq 6000, approximately 43 million clean reads of each sample were obtained with Q20 percentages of 97% or greater (**Table S2**). All clean reads were assembled by Trinity, resulting in 177195 transcripts with more than 75% of reads mapped. More than 124677 unigenes were expressed in the roots of *D. asperoides* (**Table S3**), 74463 unigenes were functionally annotated. The gene expression level was calculated based on the total mapped reads using FPKM.

### Global analysis of DEGs

**Figure 3:**
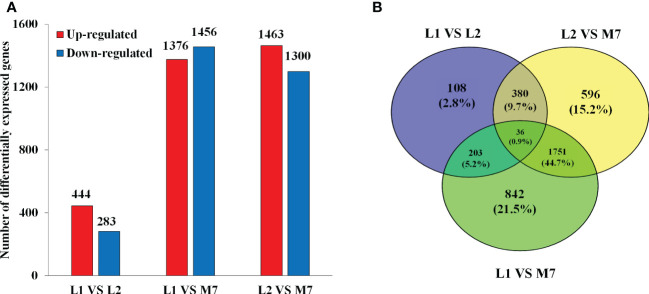
Differentially expressed genes (DEGs) in the root of *D. asperoides.* **(A)** The number of DEGs up- or down-regulated at L1, L2 and M7. **(B)** Venn diagram for the number of DEGs at different points in root of *D. asperoides*.

In the transcriptome database, a total of 27534 unigenes were detected and 3916 DEGs were obtained (**Figure 3A**). Compared with L1 and L2, there were more DEGs in M7 (**Figure 3B**). Compared with L1, 444 unigenes were up-regulated and 283 unigenes were down-regulated in L2. Only 108 unigenes were specifically differential expressed. By contrast, 1376 unigenes were up-regulated and 1456 unigenes were down-regulated in M7. Compared with L2, about 1463 unigenes were up-regulated and 1300 unigenes were down-regulated in M7. These results indicated that the content of asperosaponin VI increased and there were more DEGs as the roots developed. Thus, we believe that the biosynthesis of asperosaponin VI may regulated by DEGs in *D. asperoides*.

**Figure 4:**
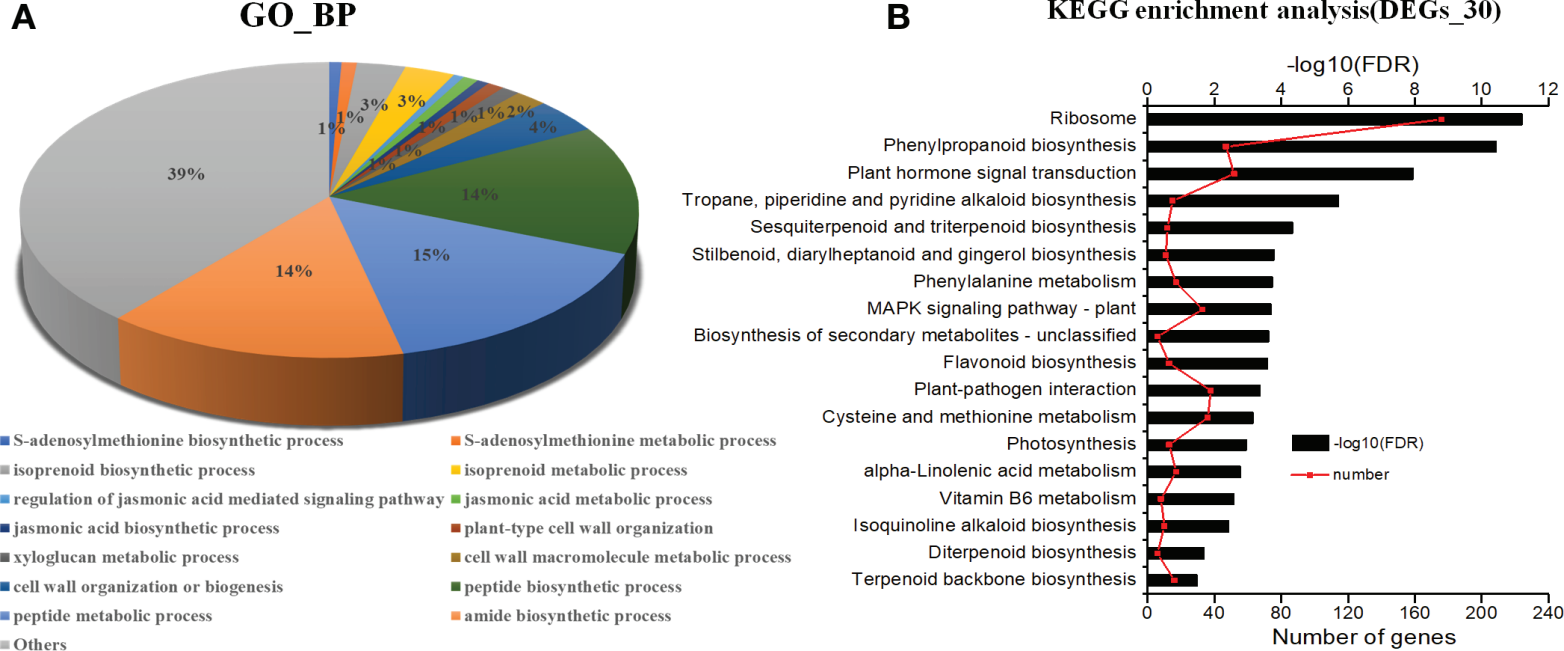
Analysis of KEGG (Kyoto Encyclopedia of Genes and Genomes) pathways and GO (Gene Ontology) enrichments. **(A)** KEGG pathways (*P <*0.001). **(B)** GO enrichments (biological processes, FDR<0.001). The data are for the functional classification of all DEGs.

The GO functional classification analysis provided useful and potential contributors to the regulatory networks in the transcriptome (**Figure 4A**). All 3916 DEGs were subjected to GO functional classification analysis to calculate the functional category based on the biological process. Most of the DEGs were assigned to 14 biological processes. Genes associated with isoprenoid biosynthetic process and isoprenoid metabolic process participated in terpenoid metabolism. Regulation of jasmonic acid mediated signaling pathway (1%), jasmonic acid metabolic process (1%), and jasmonic acid biosynthetic process (1%) were involved in the plant response to JA and abiotic stresses. The 36 DEGs that are common in all three categories enriched in 6 functional categories based on the biological process (**Table S4**). The most DEGs are response to light and may be the main factor for plant growth and development. And one of them had terpene synthase activity. 108 DEGs unique expressed in L2, including 10 genes were enriched in terpenoid biosynthetic and metabolic, 2 genes were enriched in jasmonic acid signaling. They may involve in regulation and biosynthesis of asperosaponin VI (**Figure S3A**). Therefore, the DEGs unique expressed in M7 compared with L1, approximately 103 genes enriched in cell wall metabolic, polysaccharide, xyloglucan metabolic, may relate to root development (**Figure S3B**). Moreover, the intersection between L1, M7 and L2, M7 was enriched most DEGs. Among the 1751 DEGs, 8 genes were enriched in terpene biosynthetic and metabolic, 12 genes were enriched in jasmonic acid signaling (**Figure S3C**).

The KEGG pathway analyses were performed for further functional enrichment analysis (**Figure 4B**). All of the DEGs from the three developmental stages were assigned to 18 KEGG pathways. Major pathways related to terpenoid metabolism included tropane, piperidine and pyridine alkaloid biosynthesis (15), sesquiterpenoid and triterpenoid biosynthesis (12), diterpenoid biosynthesis (6), terpenoid backbone biosynthesis (16). Several pathways were associated with phenylpropane metabolism, such as phenylpropanoid biosynthesis (47), flavonoid biosynthesis (13), and phenylalanine metabolism (17). There were several pathways related to JA metabolic pathway, such as plant hormone signal transduction (52), and α-linolenic acid metabolism (17). The results revealed that JA signal may play an important role in the biosynthesis of asperosaponin.

### Genes potentially involved in asperosaponin VI biosynthesis

Combined with GO enrichment analyses and KEGG pathways, the genes related to asperosaponin VI biosynthesis, including transcription factors, plant hormone signal transduction pathways, α-linolenic acid metabolism, JA biosynthesis and signaling, and triterpenoid biosynthesis were identified.

### Terpenoid biosynthesis

**Figure 5:**
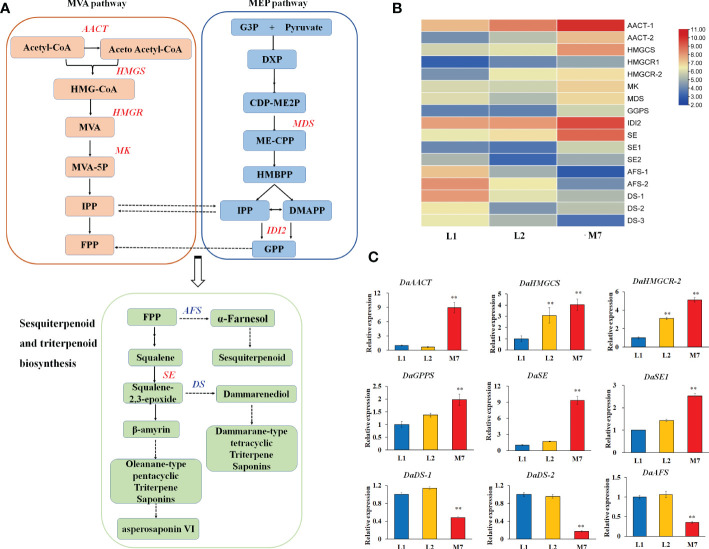
The biosynthesis of sesquiterpenoid and triterpenoid at different developmental stages. **(A)** The biosynthesis pathway of sesquiterpenoid and triterpenoid was produced according to the KEGG database. **(B)** The heat map of genes associated with terpenoid backbone, sesquiterpenoid and triterpenoid biosynthesis based on their expression level. **(C)** The expression of *DaAACT, DaHMGCS, DaHMGCR, DaGPPS, DaSE, DaSE1, DaDS-1,DaDS-2, DaAFS* in the pathway of A during root development. The values are the means ± SD; n = 3. Statistical analyses were performed using Student’s t test compared with L1. **, P < 0.01. AACT, Acetyl-CoA acetyltransferase; SE, squalene epoxidase; DS, dammarenediol synthase; AFS, alpha-farnesene synthase.

In our study, we found that most genes involved in the pathways of sesquiterpenoid and triterpenoid biosynthesis, and terpenoid backbone biosynthesis showed significant differences expression (**Figure 5A**). In our DEG data, 9 genes involved in terpenoid backbone biosynthesis, including acetyl-CoA acetyltransferase (*DaAACT*), 3-hydroxy-3- methylglutaryl coenzyme A reductase (*DaHMGCR*), 2C-methyl-D-erythritol 2,4-cyclodiphosphate synthase (*DaMDS*), and mevalonate kinase (*DaMK*), were up-regulated during the developmental stages of the roots (**Figure 5B**). Moreover, two alpha-farnesene synthase (*DaAFS*) genes related to sesquiterpenoid biosynthesis were down-regulated. In the pathway of triterpenoid saponins biosynthesis, three *DaDS* genes involved in dammarane-type triterpene saponin biosynthase were down-regulated. Furthermore, the genes in the biosynthesis of asperosaponin VI were up-regulated, including squalene epoxidase (*SE*), (**Figure 5B**). To confirm the results of transcriptomic analysis, we identified the expression of several terpenoid biosynthesis genes by qRT-PCR. The expression of **Da*AACT, DaGPPS, DaSE* and *DaSE1* in the roots of M7 was higher than in L1. The expression of *DaHMGCS*, *DaHMGCR-2* was up-regulated significantly in L2 and M7, compared with L1. Whereas *DaAFS*, *DaDS-1* and *DaDS-2* transcripts were inhibited in M7 (**Figure 5C**). These results suggested that asperosaponin VI biosynthesis pathways were activated during root development. In addition, the biosynthesis of sesquiterpenoid and dammarane-type triterpene saponins was inhibited in the roots of *D. asperoides*.

### α-Linolenic acid metabolism, JA biosynthesis and signaling

**Figure 6:**
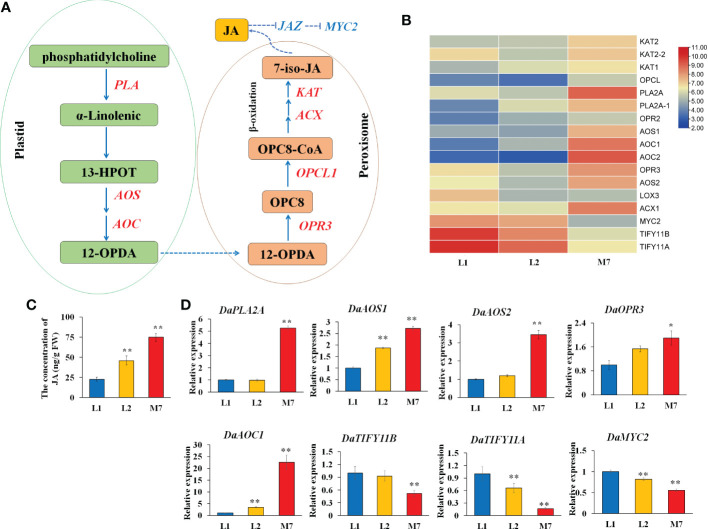
The JA biosynthesis at different developmental stages. **(A)** The pathway of α-linolenic acid metabolism and JA biosynthesis according to the KEGG database. **(B)** The heat map of genes associated with α-linolenic acid metabolism and JA biosynthesis based on their expression level. **(C)**.The concentration of JA during root development. **(D)** The expression of genes *DaPLA2A, DaAOS1, DaAOS2, DaOPR3, DaAOC1, DaTIFY11B, DaTIFY11A*, and *DaMYC2* during root development. AOS, allene oxide synthase. The values of **(C, D)** are the means ± SD; n = 3. Statistical analyses were performed using Student’s t test compared with L1. **, P < 0.01, *, P < 0.05. OPR3, 12-oxophytodienoic acid reductase; PLA2A, Phospholipase A2A.

The release of α-linolenic acid through the hydrolysis of the chloroplast membranes by phospholipases initiates JA biosynthesis (Scherer et al., 2010). In this study, several α-linolenic acid metabolism genes, JA biosynthesis and signaling related genes were differentially expressed during root development in *D. asperoides* (**Figure 6A**). A gene heatmap was illustrated based on the RNA-Seq analysis (**Figure 6B**). Genes related to α-linolenic acid metabolism and JA biosynthesis, such as two phospholipase genes (*DaPLA*), two allene oxide synthase (*DaAOS*), two allene oxide cyclase genes (*DaAOC*), and 12-oxophytodienoic acid reductase genes (*DaOPR3*, *DaOPR2*), were up-regulated during root development. JA negative regulator *JAZ* (*DaTIFY11A, DaTIFY11B*) and *DaMYC2* were down-regulated. Moreover, the concentration of JA was gradually increased during root development in *D. asperoides* (**Figure 6C**).

To further validate the expression data, we verified the expression of α-linolenic acid metabolism and JA biosynthesis genes by qRT-PCR. The expression of *DaPLA2A*, *DaAOS2* and *DaOPR3* was strongly up-regulated in the root of M7 compared with L1.And the gene expression of *DaAOS1*, *DaAOC1* in the root of L2, M7 was significantly higher than in the root of L1. In addition, JA signaling related genes *DaTIFY11A*, *DaTIFY11B*, and *DaMYC2* was significantly down-regulated expression in M7. (**Figure 6D**). The results illustrated that the α-linolenic acid metabolism and JA biosynthesis and signaling was activated during root development in *D. asperoides*. Additionally, we speculated that JA biosynthesis and signaling regulated the biosynthesis of asperosaponin VI in *D. asperoides*.

### Plant hormone signal transduction

Hormones are important in plant growth, development, and the biosynthesis of secondary metabolites. Genes involved in plant hormone signaling pathways were identified in different developmental stage roots in *D. asperoides*, including auxin, ethylene, JA, cytokinin, ABA, gibberellic acid (GA) and salicylic acid (SA) (**Figure S4**, **Table S5**). All hormone signaling pathways were predominantly induced. In IAA signaling pathways, the IAA receptor gene TIR1 and most small auxin-up RNA (SAUR) were up-regulated, while some auxin-responsive protein/indoleacetic acid-induced protein (Aux/IAA) were down-regulated during root development. Many DEGs in the JA signaling pathway, including a MYC-related gene and three TIFY genes were differentially expressed during root development. In the ABA signaling pathways, four ABA receptor genes (PYL) were up-regulated, while three serine/threonine-protein kinase SRK2 (snrk2) genes and two protein phosphatase 2C (PP2C) genes were down-regulated. Three DELLA proteins, three GID1 genes and one GID2 were up-regulated in the GA signaling pathway. The results suggested that plant hormones played complex roles in the biosynthesis of asperosaponin VI and roots development in *D. asperoides*.

### Transcription factors

Transcription factors play an important role in regulating metabolite synthesis and metabolism, as well as regulating gene expression. This study focused on the TFs in the DEGs. A total of 146 DEGs encoding TFs were identified during different developmental stages in the roots, which belonged to 22 TF families (**Table S6**). The number of down-regulated genes was significantly greater than the up-regulated genes. The results showed that 18.6% (27 in 146) of total MYBs were down-regulated in the developmental stages of the roots, as well as 5.5% (8 in 146) WRKY, and 5.5% (9 in 146) NAC TFs. About 24.1% (35 in 146) of the ethylene-responsive TFs (ERF/AP2) were differentially expressed (**Table S5**). One MYB and 1 bHLH were unique expressed in L2. And 36 TFs were unique expressed in M7, including 5 NAC, 4 AP2/ERF, 6 MYB and 3 bHLH. Expression correlation analysis indicated that 34 TFs involved in terpenoid biosynthesis, including 11 AP2/ERF and 4 B3_superfamily (**Figure S5**, **Table S8**). qRT-PCR revealed that the expression of *DaERF1* was up-regulated during root development, especially in stage M7 (**Figure S6**).

### JA biosynthesis and signaling pathway enhanced the biosynthesis of asperosaponin VI in *D. asperoides*

**Figure 7:**
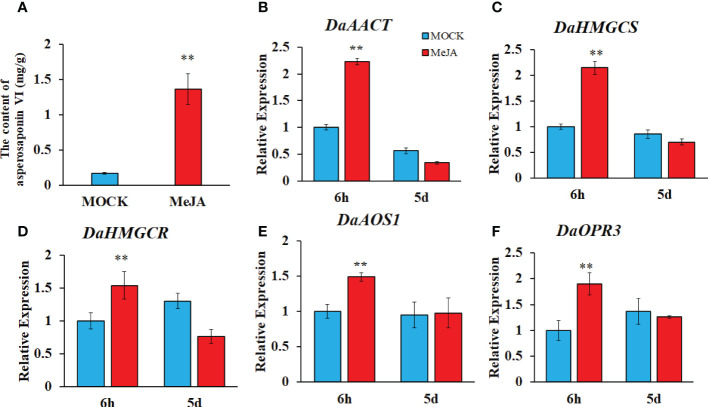
MeJA regulated the biosynthesis of asperosaponin VI in *D. asperoides.* **(A)** the content of asperosaponin VI in the roots following MeJA treatment for 5 d. **(B–F)** the expression of *DaAACT*, *DaOPR3, DaAOS1, DaHMGCS, DaHMGCR* in the roots following MeJA treatment for 6 h and 5d. All the values are the means ± SD; n = 3. Statistical analyses were performed using Student’s t test compared MOCK. **, P < 0.01.

To confirm whether the JA biosynthesis and signaling plays important role in the biosynthesis of asperosaponin VI in D. asperoides, which was subjected to MeJA treatment. The results showed that the content of asperosaponin VI was significantly accumulated following MeJA treatment in the roots of *D. asperoides* (**Figure 7A**). Moreover, the terpenoid biosynthesis gene expression of *DaAACT, DaHMGCS*, and *DaHMGCR-2* was also up-regulated after MeJA treatment for 6 h (**Figures 7B–D**). In addition, the expression of *DaOPR3*, and *DaAOS1* was up-regulated following MeJA treatment for 6 h (**Figures 7E, F**). Furthermore, the expression of *DaDS* was down-regulated. And expression level of *DaAFS* was increased following MeJA treatment (**Figure S7**). Hence, the JA biosynthesis pathway was activated. The results illustrated that JA biosynthesis and signaling regulated the expression of *DaAACT, DaHMGCS, DaHMGCR-2* and accelerated the biosynthesis of asperosaponin VI.

## Discussion

Saponins are important secondary metabolites in Chinese herbal plants, especially triterpenoid saponins. Significant difference in content of asperosaponin VI is due to different growth periods (Jiang et al., 2013). Exploring the distribution of triterpene saponins and understanding the molecular and physiological mechanisms of the synthesis and metabolism of triterpene saponins are essential in *D. asperoides*. In this study, MALDI-MSI and transcriptomics were used to analyze the distribution and mechanisms of synthesis of triterpene saponins in *D. asperoides*. The results revealed a complex gene network involving JA biosynthesis pathways and other regulatory factors involved in the biosynthesis of triterpene saponins.

### Root growth and development resulted in differences in the biosynthesis and distribution of asperosaponin VI

Secondary metabolites participate in plant growth, development, and adaptation, and may in addition play important roles in plant defense against pathogens and abiotic stress (Bednarek and Osbourn, 2009; Peng et al., 2021). Triterpenoid saponins are known as a vital class of secondary metabolites with important medicinal value (Ge et al., 2016; Yan et al., 2018). A few histochemical staining methods have been applied for the visualization of the distribution of various triterpenoid saponins, which play important roles in the efficacy of traditional Chinese medicine (Kubo et al., 1981). However, it is noteworthy that these staining methods are unable to distinguish different types of triterpenoid saponins, especially asperosaponin VI in *D. asperoides*. In this study, asperosaponin VI was visualized with MALDI MSI for the first time. Asperosaponin VI was distributed in the xylem and was enriched throughout the entire xylem from the two-leaf stage (**Figure 2H**). Thus, we speculate that the content of asperosaponin VI increased because of the increase of xylem proportion in roots during root development.

### Complex gene networks involved in the biosynthesis of asperosaponin VI

Complex secondary metabolic regulation processes exist in Chinese herbal plants. Genomes have been used to elucidate the metabolic processes in some important plants, such as *Panax ginseng* Xu et al., 2017 *Panax notoginseng* (Wei et al., 2017; Chen et al., 2018; Jiang et al., 2020), and *Tripterygium wilfordii* (Tu et al., 2020). With the rapid development of sequencing technology, transcriptome sequencing has emerged as a rapid method for researching the regulation and molecular mechanisms of complex growth and development processes, especially for *D*. *asperoides*, which lacks a reference genome. In this study, we identified 3916 DEGs (**Figure 3A**), which were enriched in the pathways of sesquiterpenoid and triterpenoid biosynthesis, terpenoid backbone biosynthesis, α-linolenic acid metabolism and plant hormone signal transduction by KEGG pathway analysis (**Figure 4B**). Herein, the DEGs were abundant in jasmonic acid metabolic process and jasmonic acid biosynthetic process (**Figure 4A**). Moreover, the unique expressed genes in L2 stage enriched in terpenoid biosynthetic and metabolic, jasmonic acid signaling (**Figure S3A**). The results indicated that JA biosynthesis and signaling, sesquiterpenoid and triterpenoid biosynthesis genes, terpenoid backbone biosynthesis genes, play an important role in regulating the biosynthesis of asperosaponin VI.

### TFs involved in JA biosynthesis and signaling and the biosynthesis of asperosaponin VI

Transcription factors play important roles in regulating plant growth and metabolite synthesis by regulating the expression of downstream genes (Hoang et al., 2017; Long et al., 2019). In this study, 146 TFs exhibited significant differences in expression. These TFs were classified into 20 families, including *ERF*/*AP2*, *MYB*, *NAC*, and *WRKY*, which indicated the regulation of metabolites synthesis in *D. asperoides* (**Tables S6**, **S7**). Expression correlation analysis that 11 AP2/ERF involved in terpenoid biosynthesis (**Figure S5**, **Table S8**). Previous studies have demonstrated that TFs function in regulating terpenoid synthesis. Tanshinone production was significantly increased in *SmERF1L1* overexpressed roots in *Salvia miltiorrhiza*. Additionally, *SmERF1L1*, a novel JA -responsive gene, positively regulated tanshinone biosynthesis by comprehensively upregulating tanshinone biosynthetic pathway genes (Huang et al., 2019). Moreover, the over-expression of *SmWRKY1* significantly increased tanshinone production in *S. miltiorrhiza*. *SmWRKY1* was responsive to MeJA and acted as a positive regulator to regulate tanshinone biosynthesis through activating *SmDXR* in the MEP pathway (Cao et al., 2018). In addition, a *R2R3*-*MYB* TF, *SmMYB98*, promoted the accumulation of tanshinone by activating the transcription of *SmGGPPS1*, *SmPAL1*, and *SmRAS1* (Hao et al., 2020). Therefore, many TFs may be regulated by JA biosynthesis and signaling. And studies on TFs will be beneficial for illustrating the regulatory networks for the biosynthesis of triterpenoids, including asperosaponin VI.

### JA biosynthesis and signaling promotes the biosynthesis of asperosaponin VI by activating the expression of triterpenoid biosynthetic genes

The phytohormone JA regulates plant development and defense processes, such as by synthesizing secondary metabolites to resist biotic or abiotic stresses (Browse, 2009; Zhou et al., 2016; Ho et al., 2020). There are some molecular links between JA biosynthesis, signaling and saponin biosynthesis. A JA biosynthetic 13-lipoxygenase gene, *PgLOX6*, promotes the production of ginsenoside, which is a triterpenoid saponin. Furthermore, *PgLOX6* up-regulates the expression of ginsenoside biosynthetic genes such as squalene synthase (*SS1*) and squalene epoxidase (*SE*) (Shadi et al., 2016). The JASMONATE ZIM DOMAINs (JAZs) are repressors in the JA signaling pathway (Bai et al., 2011), and *SmJAZ8* deregulates the yield of tanshinone in “*Danshen”* (Pei et al., 2018). JAs are formed from α -linolenic acid of chloroplast membranes by the lipoxygenase pathway (Wasternack and Song, 2016) (**Figure 6A**). In this study, the concentration of JA was increased (**Figure 6C**). Meanwhile, MeJA facilitated the accumulation of asperosaponin VI (**Figure 7A**). JA biosynthesis and signaling has a positive effect on the regulation of asperosaponin VI biosynthesis. In addition, the gene *DaAACT*, *DaHMGCS, DaHMGCR* in the pathway of saponin biosynthesis was rapidly up-regulated following MeJA treatment (**Figures 7B–D**), as well as in the developing roots. Hence, we suspect that JA biosynthesis and signaling may regulate the biosynthesis of asperosaponin VI by regulating the expression of triterpenoid biosynthetic genes including *DaAACT*, *DaHMGCS, DaHMGCR*. *DaAACT* is the first enzyme in the terpenoid synthesis pathway and catalyzes two units of acetyl-CoA into acetoacetyl-CoA. The application of AACT could increase the fraction of 3-hydroxyvalerate in *Escherichia coli* (Jeon et al., 2017). Therefore, we speculate that JA biosynthesis and signaling regulates the expression of TFs which active the expression of triterpenoid biosynthetic genes, thereby facilitating the biosynthesis of asperosaponin VI in *D*. *asperoides*.

## Conclusion

**Figure 8:**
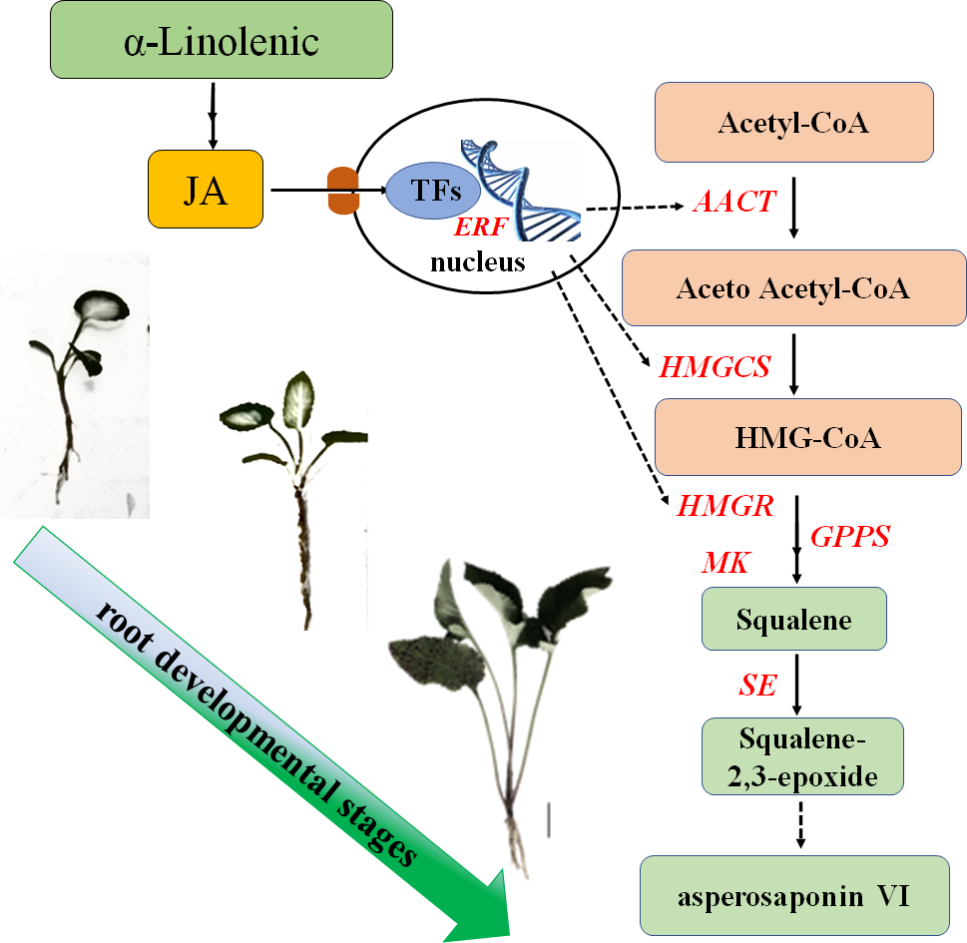
Schematic showing the role of α-linolenic acid and JA in the biosynthesis of asperosaponin VI during the root deveopmental in *D. asperoides.*.

In this study, we found that the content of asperosaponin VI and JA in the root increased gradually along with the development of the roots. Additionally, the spatial distribution of asperosaponin VI was localized in the xylem. A total of 3916 DEGs and 146 TFs were identified. The genes associated with JA biosynthesis, signaling and asperosaponin VI biosynthesis were up-regulated following MeJA treatment, as well as in the developing roots of *D. asperoides*. We speculate that JA biosynthesis and signaling regulates the expression of TFs to promote the expression of triterpenoid biosynthetic genes and facilitate the biosynthesis of asperosaponin VI (**Figure 8**). These results present a regulatory network by which asperosaponin VI, JA, and TFs co-modulate the biosynthesis of asperosaponin VI in *D. asperoides*.

## Data availability statement

The original contributions presented in the study are publicly available. The data presented in the study are deposited in the the National Center for Biotechnology Information (NCBI) database, accession number GSE208123.

## Author contributions

JX, TZ conceived and designed the study. JX wrote the manuscript. XO, YY, CX, WJ revised the manuscript. JX, ZH and CY performed the experiments. JX, HH and LL contributed to data analysis. All authors discussed the results and agreed to the published version of the manuscript.
