# Supplementary material for: Transcriptome analysis reveals that jasmonic acid biosynthesis and signaling is associated with the biosynthesis of asperosaponin VI in Dipsacus asperoides
Source: Front Plant Sci. 2022 Dec 22;13:1022075. doi: 10.3389/fpls.2022.1022075 (PMC9928152; doi:10.3389/fpls.2022.1022075)

**Transcriptome analysis reveals that** **jasmonic acid signaling is associated with the biosynthesis of** **asperosaponin VI in *Dipsacus asperoides***

Fig. S1 The standard and sample original peak map of HPLC in Fig. 2F. A-D, the sample of L1, L2, L4, and standard. E-F, the sample of M7 and standard. G-H, the sample of M12 and standard. The red arrows represent the peak of asperosaponin VI.


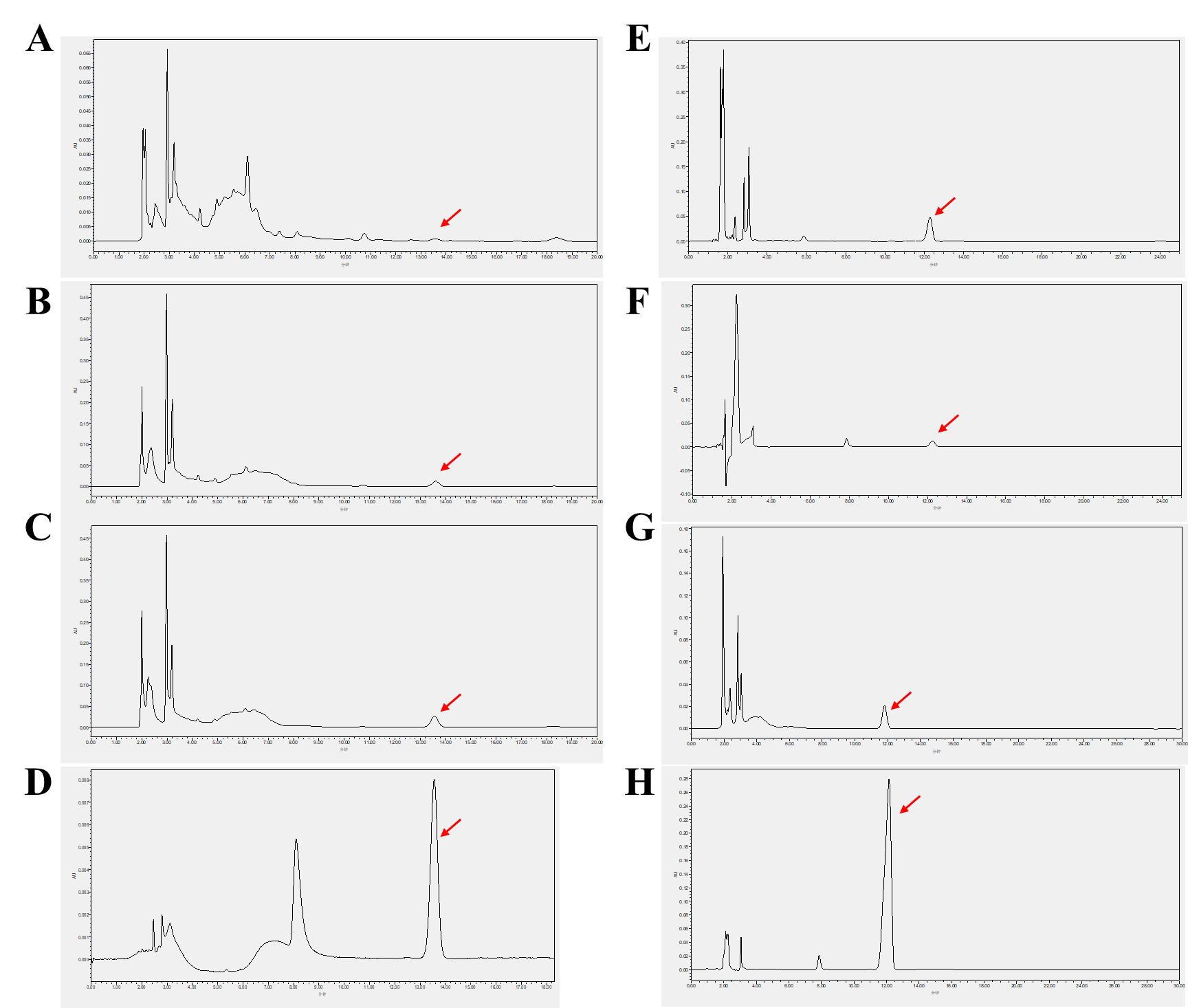


Fig. S2 The cross section of root in L2 stage.


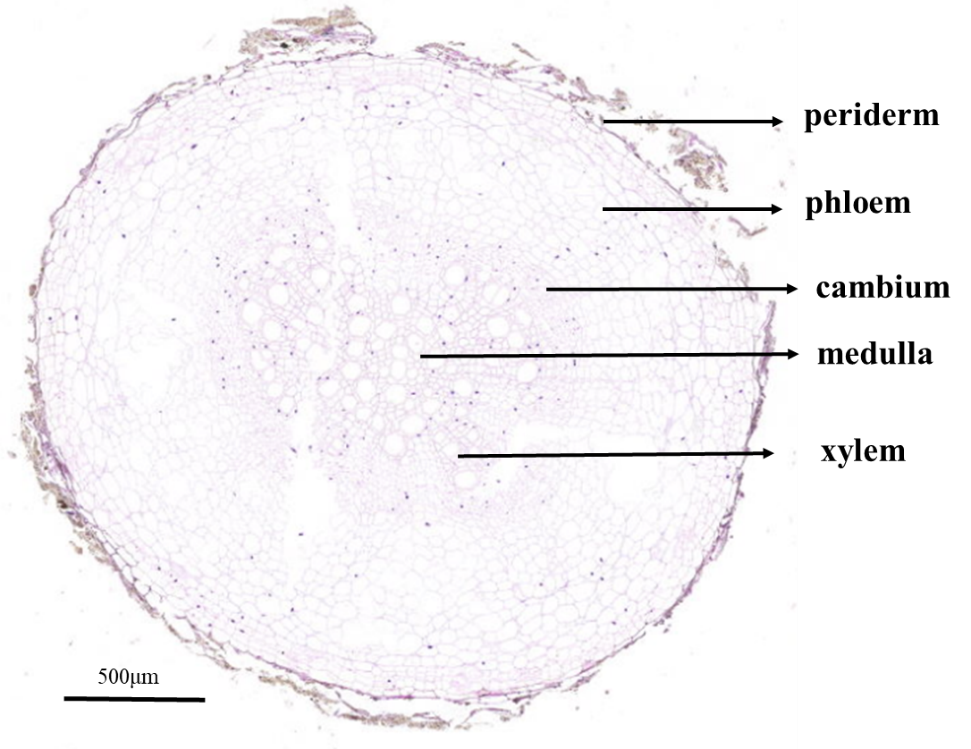


Fig. S3 GO enrichments. A, GO enrichments of L2 unique genes. B, GO enrichments of M7 unique genes. C, GO enrichments of intersection between L1 VS M7 and L2 VS M7.


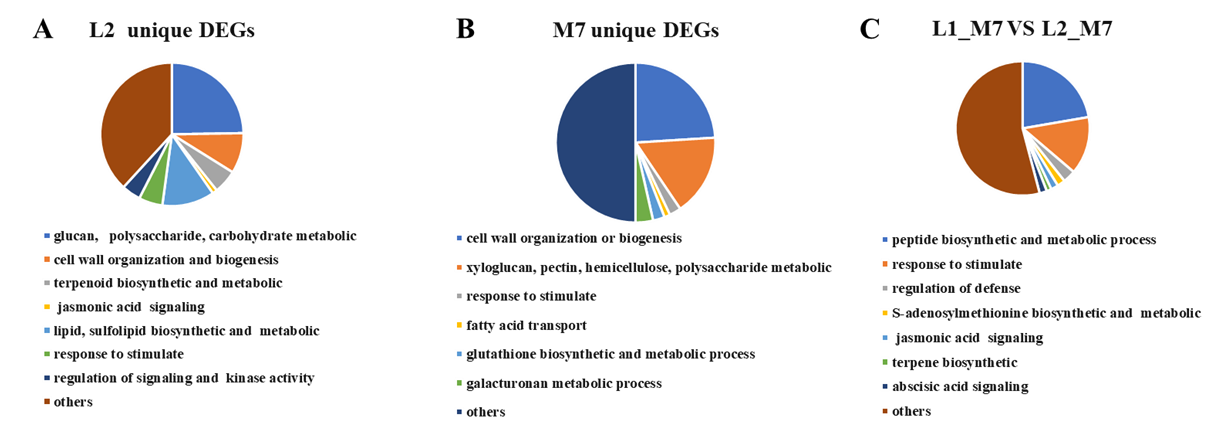


Fig. S4 The expression patterns of genes in plant hormone signal transduction pathway with heat map.


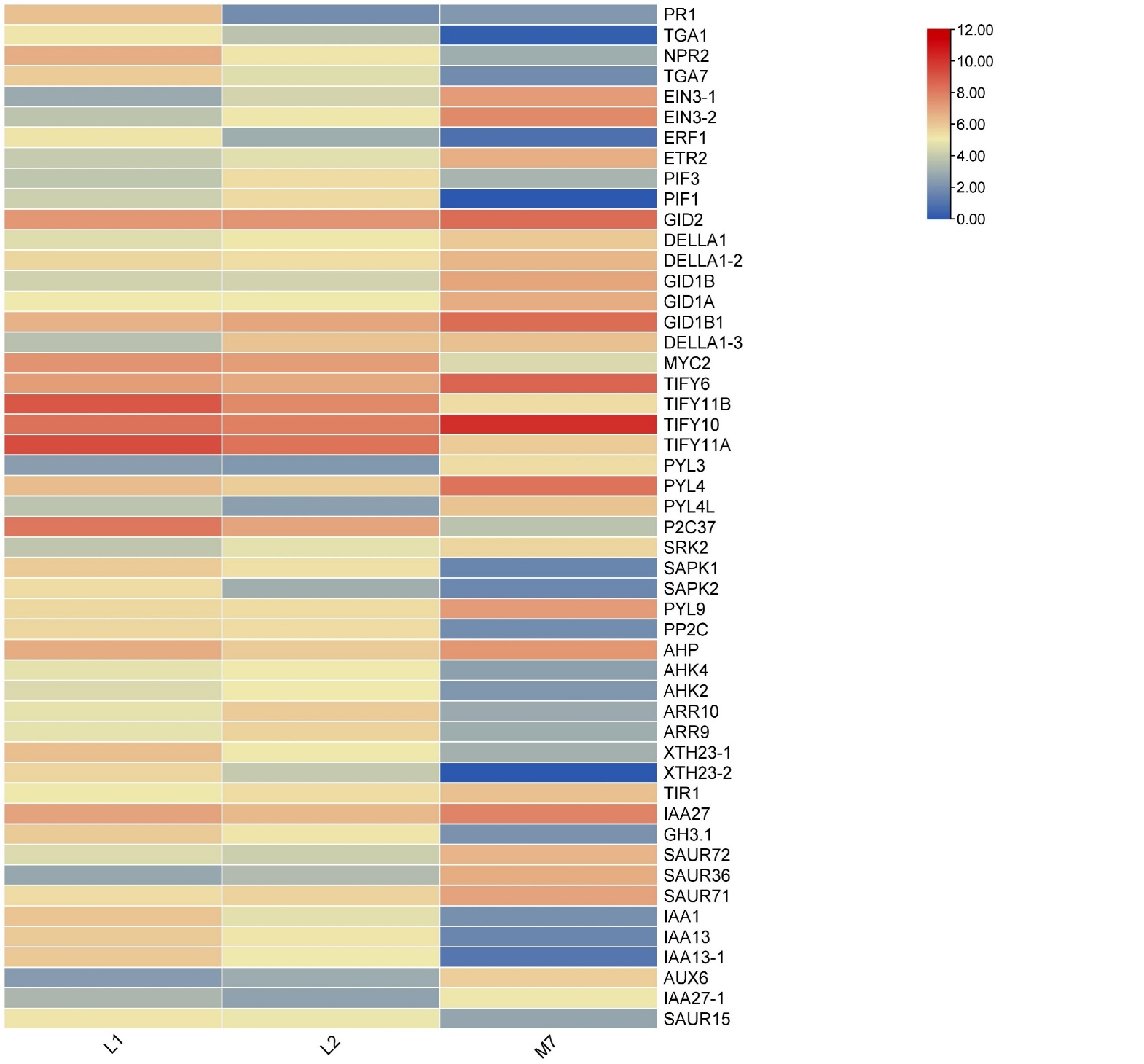


**SA**

**ETH**

**GA**

**JA**

**ABA**

**cytokinin**

**IAA**


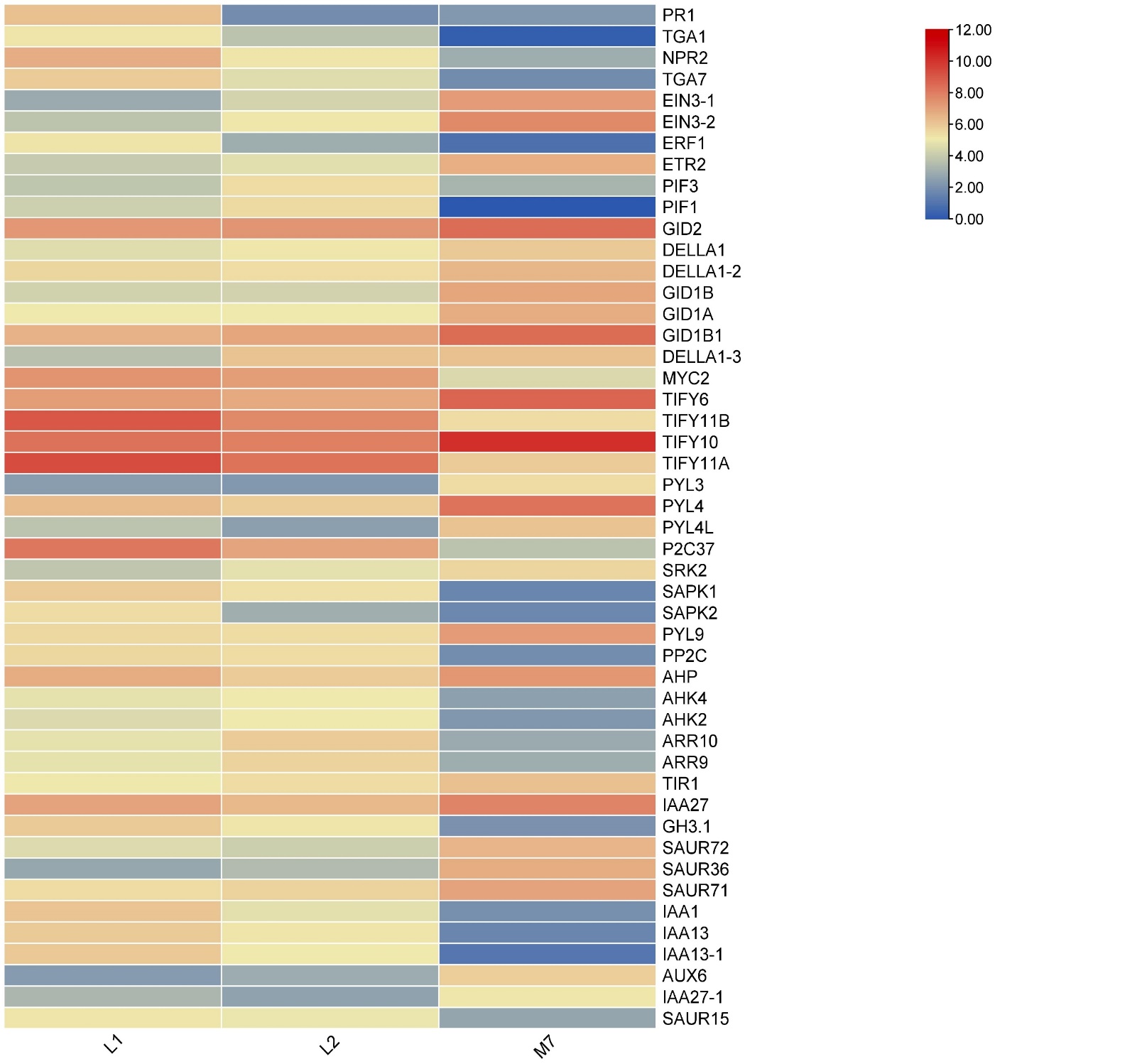

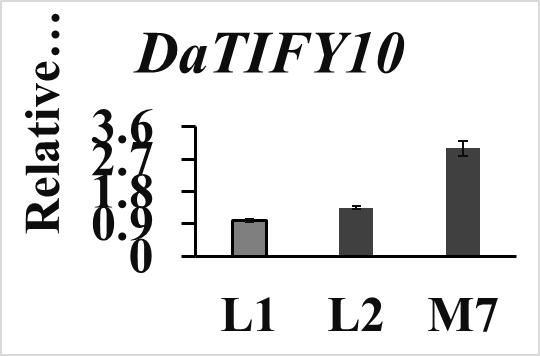

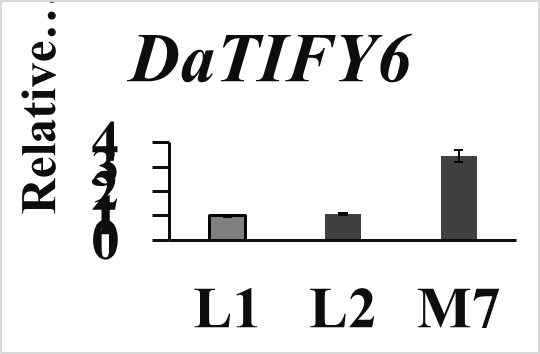

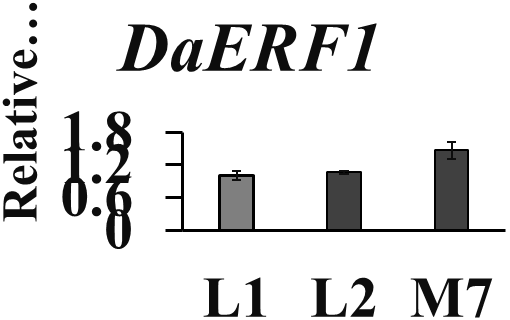


**L1**

**L2**

**M7**

**A**

**B**

******

******

*****


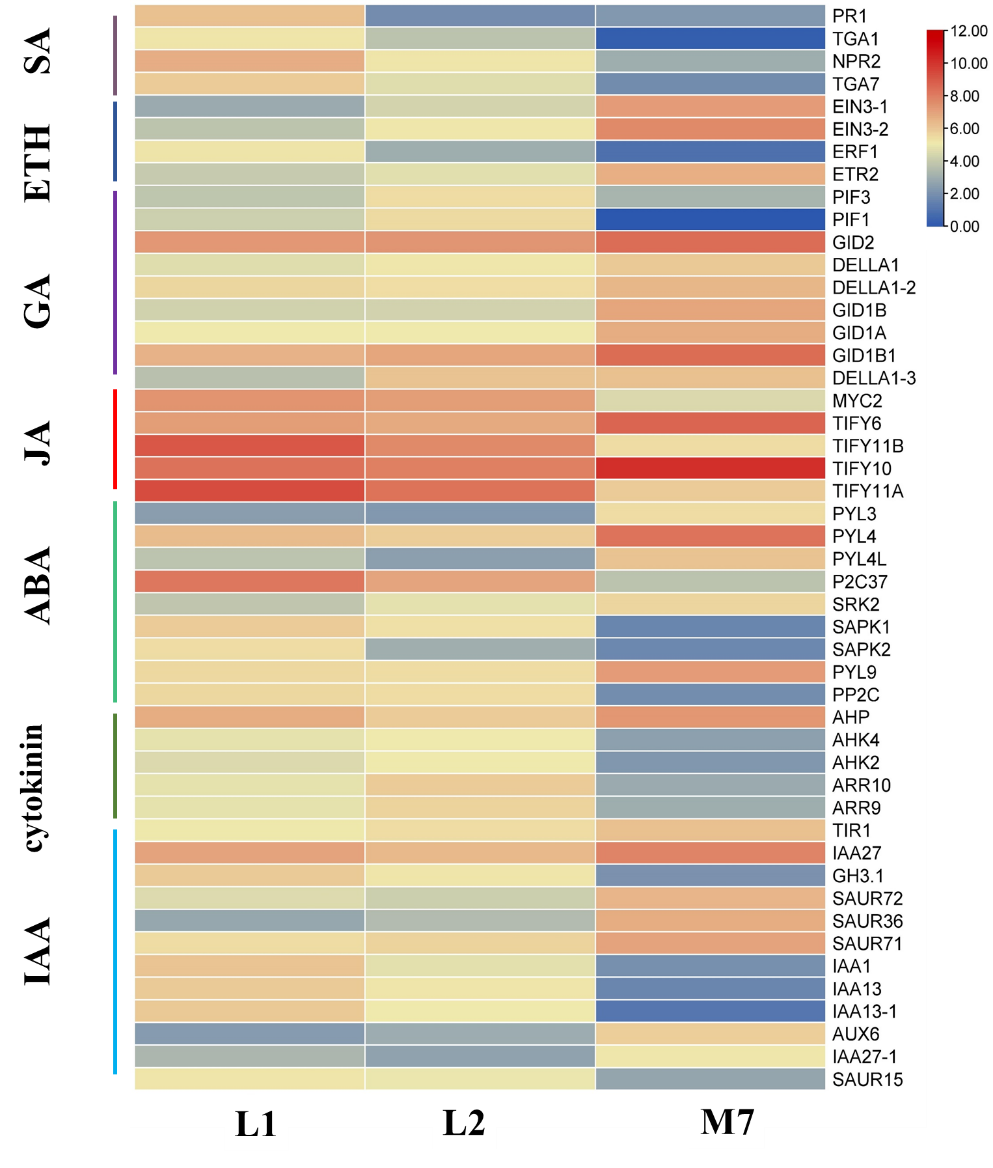


Fig. S5 The correlation coefficients between TFs and terpenoid biosynthesis genes. Red dots are terpenoid biosynthesis genes and blue dots are TFs.


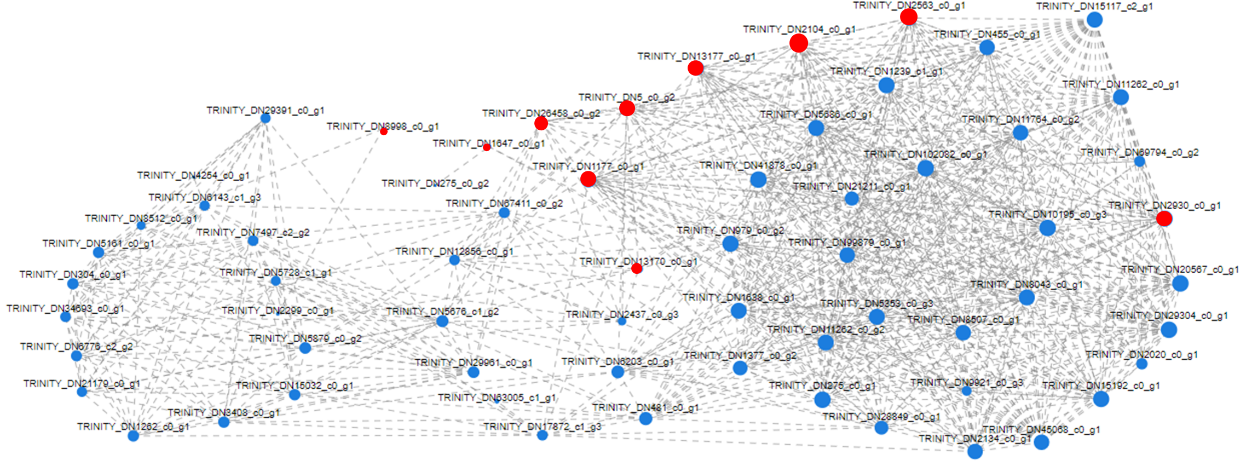


Fig. S6 The expression of genes *DaERF1* during root development. Statistical analyses were performed using Student’s t test compared L1. *, P < 0.05.


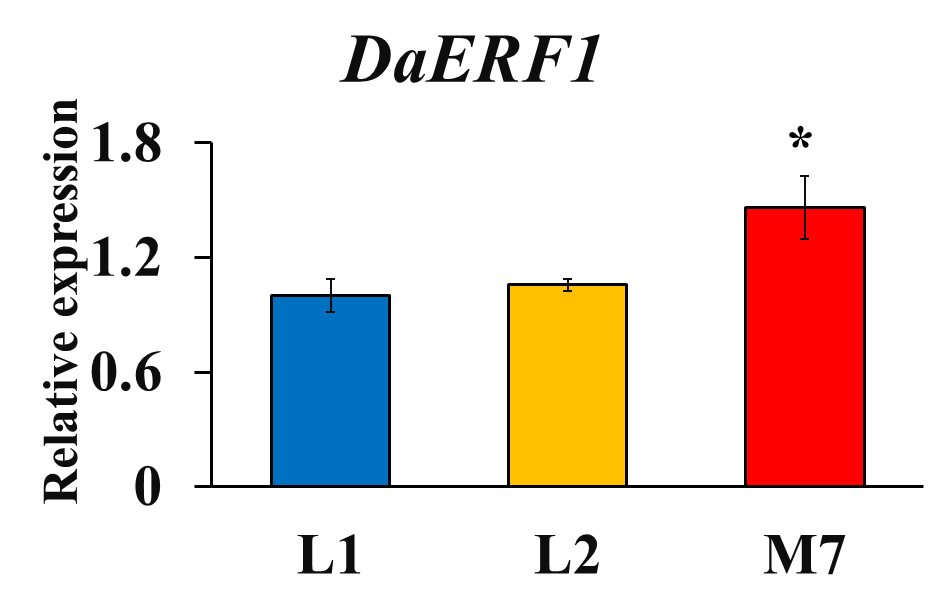


Fig. S7 MeJA regulated the biosynthesis of terpenoid in *D. asperoides.*


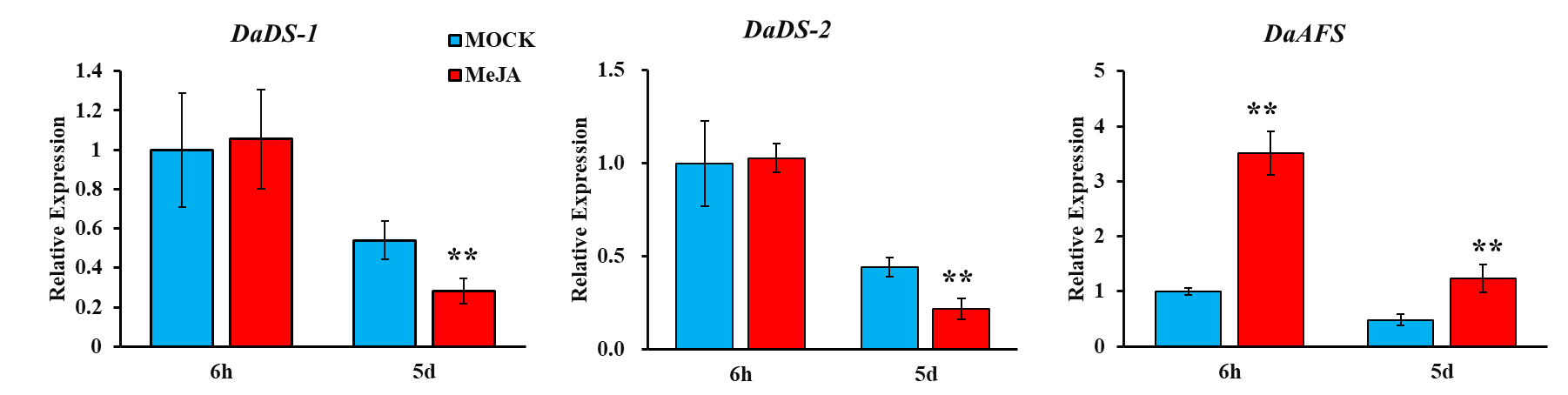

Supplement: Supplementary file 2 [file DataSheet_1.docx]
